# Defining the Magnetic Resonance Features of Renal Lesions and Their Response to Everolimus in a Transgenic Mouse Model of Tuberous Sclerosis Complex

**DOI:** 10.3389/fonc.2022.851192

**Published:** 2022-06-23

**Authors:** Shubhangi Agarwal, Emilie Decavel-Bueff, Yung-Hua Wang, Hecong Qin, Romelyn Delos Santos, Michael J. Evans, Renuka Sriram

**Affiliations:** ^1^ Department of Radiology and Biomedical Imaging, University of California, San Francisco, San Francisco, CA, United States; ^2^ Department of Pharmaceutical Chemistry, University of California, San Francisco, San Francisco, CA, United States; ^3^ Helen Diller Family Comprehensive Cancer Center, University of California, San Francisco, San Francisco, CA, United States

**Keywords:** TSC, kidney, everolimus, mTOR, MRI, mp-MRI, AML

## Abstract

Tuberous sclerosis complex (TSC) is an inherited genetic disorder characterized by mutations in *TSC1* or *TSC2* class of tumor suppressers which impact several organs including the kidney. The renal manifestations are usually in the form of angiomyolipoma (AML, in 80% of the cases) and cystadenomas. mTOR inhibitors such as rapamycin and everolimus have shown efficacy in reducing the renal tumor burden. Early treatment prevents the progression of AML; however, the tumors regrow upon cessation of therapy implying a lifelong need for monitoring and management of this morbid disease. There is a critical need for development of imaging strategies to monitor response to therapy and progression of disease which will also facilitate development of newer targeted therapy. In this study we evaluated the potential of multiparametric ^1^H magnetic resonance imaging (mpMRI) to monitor tumor response to therapy in a preclinical model of TSC, the transgenic mouse A/J *Tsc2^+/-^*. We found 2-dimensional T_2_-weighted sequence with 0.5 mm slice thickness to be optimal for detecting renal lesions as small as 0.016 mm^3^. Baseline characterization of lesions with MRI to assess physiological parameters such as cellularity and perfusion is critical for distinguishing between cystic and solid lesions. Everolimus treatment for three weeks maintained tumor growth at 36% from baseline, while control tumors displayed steady growth and were 70% larger than baseline at the end of therapy. Apparent diffusion coefficient, T_1_ values and normalized T_2_ intensity changes were also indictive of response to treatment. Our results indicate that standardization and implementation of improved MR imaging protocols will significantly enhance the utility of mpMRI in determining the severity and composition of renal lesions for better treatment planning.

## Introduction

Tuberous sclerosis complex (TSC) is an autosomal dominant syndrome caused by germline inactivating mutations in either allele of the genes *TSC1* or *TSC2*. The *TSC1/TSC2* tumor suppressor gene complex, also known as the Hamartin and Tuberin protein complex, negatively regulates mechanistic target of rapamycin (mTOR) complex 1 (mTORC1), a master regulator of cellular biosynthesis, resulting in proliferation, angiogenesis and uncontrolled cell growth. This disorder affects multiple organ systems and the clinical manifestations of TSC include tumors in brain, skin, heart, lungs, and kidneys and neurological conditions such as seizures, autism, and cognitive disability. The renal manifestations are usually in the form of angiomyolipoma (AML, in 70-80% of the cases) and cystadenomas and are one of the main causes of mortality and morbidity in patients with TSC (1). The severity of renal involvement is markedly increased in disease caused by *TSC2* compared to *TSC1* mutations (2). TSC renal cysts have a range of disease patterns and severity reflected by a clinical scoring system that has been developed independent of the Bosniak scale (3). These lesions are under-recognized for causing severe disease which manifests as hypertension and chronic kidney disease. Management of TSC cystic disease is not well-studied although controlling blood pressure (4) and treating with mTOR (5) inhibitors have shown benefit in reducing cystic burden.

Unlike sporadic AML tumors that are unilateral and smaller in size, those associated with TSC are multiple, bilateral, and asymptomatic. However, as they progress to larger lesions (>3 cm), they run the risk of bleeding and require prophylactic management to prevent renal impairment which can sometimes be fatal. Transarterial embolization of these lesions at high risk for bleeding has been shown to slow tumor growth and preserve renal function (6). However, repeat treatment is often required and necessitates regular radiographic follow-up. Thus, constant monitoring after diagnosis, for tumor growth and emergence of new tumors, is implicit in the management of TSC-associated AML.

mTOR pathway inhibitors such as everolimus and sirolimus are the first line of therapy against asymptomatic AML (7). Studies have shown that early treatment prevents the progression of AML and can in fact cause tumor shrinkage, but tumors regrow upon cessation of therapy, implying a lifelong need for monitoring and management. Therefore, imaging assessment of disease prevalence and treatment response is essential for management of this disease. Magnetic resonance imaging (MRI) is the recommended modality to follow these renal lesions that often have cystic components and are sometimes fat-poor (8).

The *Tsc2* ^+/-^ A/J mouse is heterozygous for deletion of exons 1-2 and is considered a good model to study TSC-related kidney disease because the mice develop age-related renal cysts and kidney tumors (cystadenomas and cystadenocarcinomas) with a defective mTOR pathway like that observed in human TSC-related tumors (9–11). This model has proven invaluable for evaluating numerous therapies (12–14) but the lack of longitudinal noninvasive measures of treatment effect is a significant barrier to full utilization of this model. In fact, this model has not been characterized with imaging modalities such as MRI which is an indispensable tool in the clinical workup of patients with TSC. Hence, the goal of this pilot study was to evaluate and optimize MR imaging protocols for monitoring renal lesions and to assess the potential of multiparametric ^1^H MRI (mpMRI) to monitor tumor response to therapy in this preclinical transgenic mouse model of TSC.

## Materials and Methods

### Animal Model

Six male A/J strain *Tsc2* ^+/-^ mice (courtesy Tuberous Sclerosis Complex Alliance), 6-7 months old, were studied (n = 3 control and n = 3 treatment). All procedures were approved by our Institutional Animal Care and Use Committee. The mice were treated with everolimus (RAD001, Sigma-Aldrich), 5 mg/kg administered intraperitoneally daily for a total of 3 weeks. Everolimus was dissolved in PEG 400 and 20% 2-hydroxypropylcylcodextrine in water + dimethylsulfoxide (DMSO, Sigma-Aldirch).

### ^1^H MR Imaging

Tumor-bearing mice were imaged using a vertical wide bore 14.1T scanner (1,000 mT/cm gradients, Agilent) equipped with a millipede 40 mm ^1^H coil for anatomic imaging. Mice were anesthetized with 1–2% inhalant isoflurane. Multiparametric imaging was performed which included T_2_-weighted imaging for morphology, diffusion weighted images for cellularity, T_1_-weighted images with variable flip angle for T_1_ mapping followed by dynamic contrast enhanced (DCE) imaging for measuring perfusion. High resolution T_2_-weighted images were acquired for anatomic references using a fast spin echo sequence with fat suppression and the following parameters: field of view, 30 x 30 mm; matrix size, 256 x 256; repetition time, 3 s; echo time, 10 ms; ETL 8; segments 32; NEX, 2; slice thickness 1 and 0.5 mm, interleaved acquisition in both axial and coronal orientations. High resolution 3D images were acquired using a fast spin echo sequence and the following parameters: repetition time, 200 ms; echo time, 9 ms; field of view, 30 x 30 mm; slab thickness, 64 mm; matrix size, 256 x 256 x 64 (128 for 0.5 mm thick slices); NEX, 2; slice thickness 0.5-mm. Diffusion weighted images with respiratory gating were acquired using the following parameters: matrix size, 128 × 128; field of view, 30 × 30 mm; slice thickness, 1 mm; *b*-values of 25, 180, 323, 508 s/mm^2^. T_1_ mapping was performed by acquiring gradient echo images with field of view, 30 x 30 mm; matrix size, 128 x 128; repetition time, 39 ms; echo time, 3 ms; slice thickness 1 mm with the following flip angles: 2, 5, 10, 15, 20, 30 and 40 degrees. Following T_1_ mapping, a bolus of Gd-DTPA (0.27 mmol/kg, Magnevist, Bayer Healthcare, Whippany, NJ) was injected *via* the tail vein followed by 150 µL of saline flush. DCE MRI was performed with the following parameters: TE/TR =1.11/39 ms, 40° flip angle, 128´128 matrix, 30 x 30 mm FOV, 0.3125 x 0.3125 mm in-plane resolution, 1 mm slice thickness, 40 dummy scans (prior to contrast agent injection), and 5s temporal resolution with total 50 time points. After baseline imaging, the three mice were either treated with everolimus and the remaining three were untreated controls. All the six mice were imaged weekly with the same imaging protocol for a total of three weeks.

### Data Analysis

All image processing and analysis were performed using MATLAB (Mathworks, Natick, MA, USA), and IDL based in-house software BRIMAGE. All the data presented in this study is from 3 mice per cohort. T_2_-weighted images were used to calculate the volume of tumor lesions in mice and were tracked over time by manually drawing regions of interest (ROIs) around them. T_2_-weighted intensities for all lesions were calculated and normalized to blood vessel intensity to evaluate the serial changes and across groups. We normalized the T_2_-weighted intensities to blood vessel intensity as we do not expect the latter to be affected by age and/or treatment. Apparent diffusion coefficient (ADC, mm^2^/s) maps were generated as previously described (15) and mean tumor ADCs for the same ROIs. Briefly, ADC were estimated on the basis of mono-exponential fitting of diffusion-weighted signal of 4 b-values to the equation *S = S0•exp(–ADC• b)* using VNMRJ software (Agilent Technologies). Baseline T_1_ maps and semi-quantitative analysis of DCE data was performed using the techniques previously described (16). We looked at the following semi-quantitative parameters based on dynamic Gd-DTPA concentration: 1) area under the curve (AUC): sum of Gd-DTPA concentrations at all time points; 2) initial area under the curve (iAUC): sum of Gd-DTPA concentration from contrast agent arrival to 90s after the arrival; 3) wash-in slope: approximate derivative of dynamic Gd-DTPA concentration curve from bolus arrival to the peak; 4) wash-out slope: the slope of linear regression of Gd-DTPA concentration from the time of peak enhancement to the last time point, with positive slopes allowed; 5) time to reach peak concentration and 6) peak concentration. We also evaluated the quantitative parameter K^trans^ (volume transfer constant) that represents the permeability using the Tofts model (16).

### Statistical Analysis

Data are presented as mean ± standard error. Statistical analysis was performed using one- and two-way ANOVA and linear mixed model. One-way ANOVA was performed on total tumor burden, ADCs, normalized T_2_ intensities and T_1_ values of each group at baseline. A repeated measures one-way ANOVA with Tukey’s *post hoc* tests was used to assess the impact of treatment on the two cohorts for the multiple imaging parameters. P-values < 0.05 were considered statistically significant. Statistical tests were performed using PRISM (GraphPad, La Jolla, CA, USA) and Stata 16 (StataCorp LLC, College Station, TX). Changes in imaging parameters of a lesion over time between treated and control mice were assessed *via* multilevel regression analyses. The linear mixed model analysis was conducted using ‘mixed’ function in Stata 16. Time was used as a continuous variable, and the overall linear trends in imaging parameters after therapy were obtained and reported.

### Immunohistochemistry

Kidneys were harvested after the last imaging session at the end of the third week of treatment. Left and right kidneys were formalin-fixed and paraffin-embedded for histopathological analysis. Fixed kidney blocks were cut into 4 µm-thick sections on a Leica microtome (Buffalo Grove, IL, USA) in coronal orientation, then stained with hematoxylin and eosin (H&E), and Ki67/Rabbit antibody #9129 (Cell Signaling Technology Europe, B.V.). Brightfield images of lesions stained with H&E and Ki67 were acquired using a Nikon 6D microscope using a 40× power objection yielding a 0.22 μm in-plane resolution to focus on the lesions. Stitched images of the entire kidneys were acquired at 10x magnification to assess the distribution and properties of lesions. The lesions were sub-divided into three groups: cystic (filled with ≤ 25% cells), papillary (filled with > 25% cells) and solid lesions (filled with cells) (17). Images were visualized using the open-source software QuPath (18).

## Results

### Optimal Detection of Renal Lesions Using T_2_-Weighted Images

**Figure 1:**
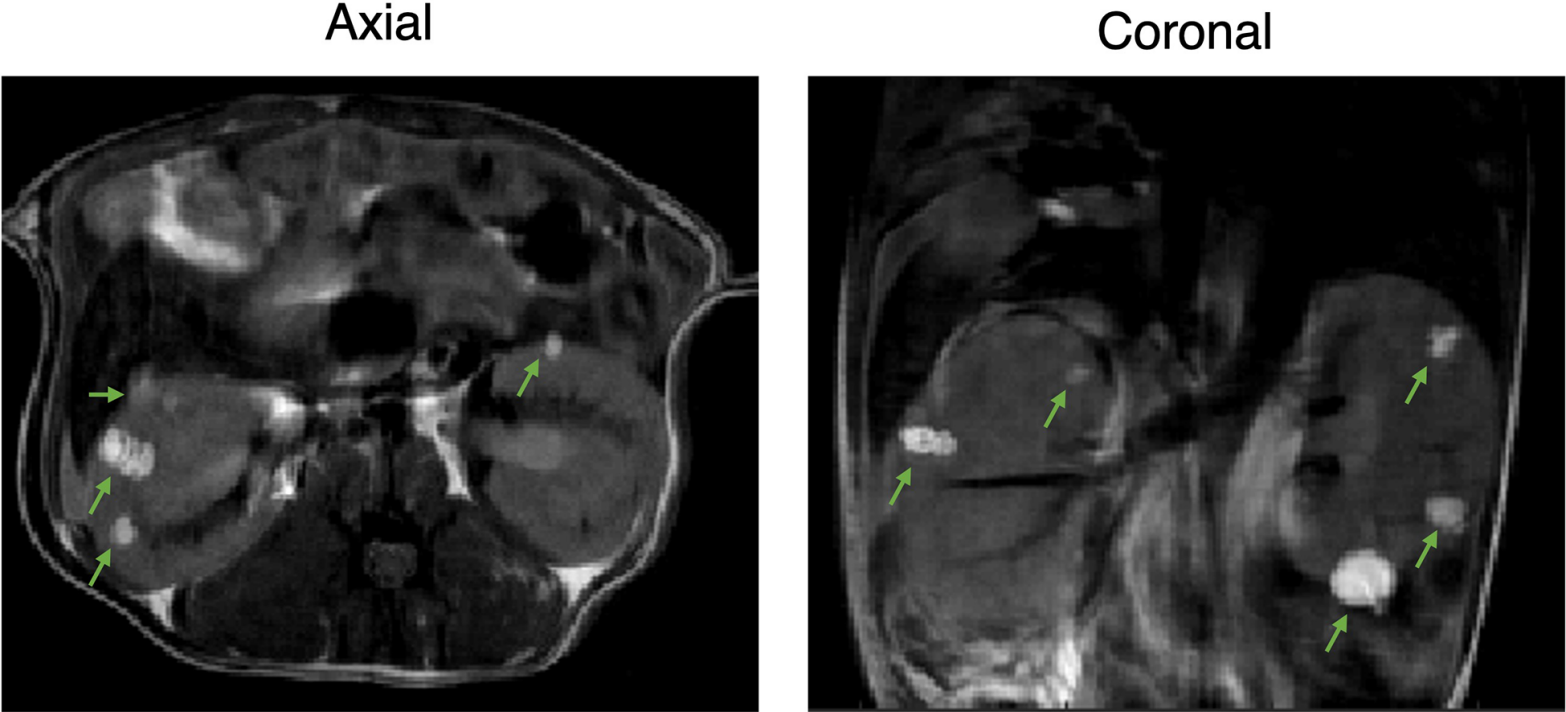
Two-dimensional proton imaging of 6–7-month-old Tsc2 ^+/-^ A/J male mice. Representative 2D T_2_-weighted images of the TSC mice with bilateral adenomas and cystic lesions in the renal capsule and pelvis at axial and coronal orientation. Lesions are indicated with green arrows.

Male *Tsc2* ^+/-^ A/J mice, 6-7 months old, presented with bilateral adenomas and cystic lesions in the renal capsule and pelvis as shown in the representative images in **Figure 1**. T_2_-weighted images were used to delineate the lesions and estimate their volumes. Tumor volumes were measured once a week for a total of four weeks. Interestingly, the majority of the lesions were observed around the renal peripheral capsule or pelvis (**Figure 1**). The average lesion size at this age was 0.4 ± 0.51 mm^3^. The lesion size ranged from 0.016 to 5.12 mm^3^ and 36 ± 5.7 lesions were detected on average in each mouse.

**Figure 2:**
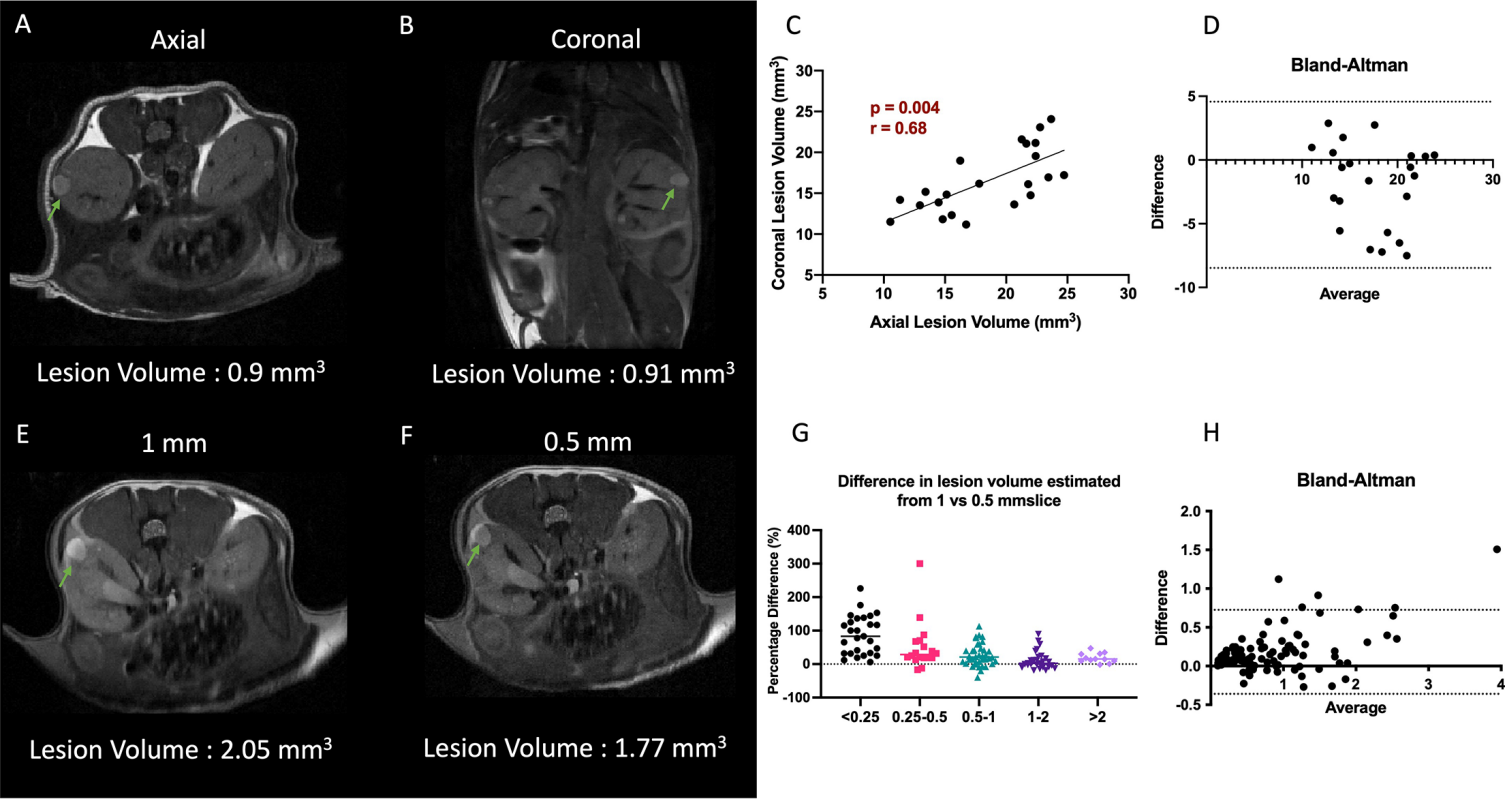
Impact of slice orientation and thickness on volume estimation. **(A, B)** Representative T_2_-weighted axial and coronal images. **(C)** Comparison of axial vs coronal slice orientation and its effect on the tumor volume estimation. **(D)** Bland-Altman plot of differences and averages from axial and coronal lesion volumes. **(E, F)** Representative T_2_-weighted 1 mm and 0.5 mm slice thickness images. Lesions are highlighted with arrows. **(G)** Decrease in difference of volumes of lesion estimated from 0.5 and 1 mm thick slices as function of lesion volume. **(H)** Bland-Altman plot of differences and averages of lesion volumes from 1 mm and 0.5 mm thick images. Lesions are indicated with green arrows.

Owing to the early assessment of developing lesions and their locations, and the chosen slice thickness of 1 mm (in order to achieve high SNR in a short time), we investigated whether volumes derived from axial or coronal imaging sections were different. Representative T_2_-weighted images of the abdomen acquired at axial and coronal orientations in shown in **Figures 2A, B**. **Figure 2C** shows the Pearson correlation analysis between the mean tumor volumes with a highly significant positive correlation (p= 0.004, r = 0.68). A Bland-Altman analysis of differences and agreement between the volumes showed that the majority of lesion volumes were well within the lines of agreement (**Figure 2D**).

However, considering that the majority (>90%) of the lesions were below 2 mm^3^, we investigated if 0.5 mm thick imaging slices would yield a better estimate of the lesion volume and minimize the partial volume effect. For this analysis the mice were imaged in the axial orientation consecutively with 0.5 as well as 1 mm slice thickness interleaved to cover the entire kidney. A total of 122 lesions were identified in both data sets. Representative T_2_-weighted images with 1 mm and 0.5 mm slice thickness are shown **Figures 2E, F**. A highly significant positive correlation (p<0.0001, r = 0.97) was obtained between the tumor volumes obtained by the two methods (data not shown). On average the lesion volumes estimated by 0.5 mm thick slice (1 ± 1.18 mm^3^) were ~20% lower than when measured by 1 mm slice thickness T_2_-weighted sequence (1.2 ± 1.3 mm^3^). Analysis of the difference in volumes per lesion from the two different slice thickness images showed that 1 mm thick images were overestimating the volumes significantly more for smaller lesions than larger lesions (p<0.001, **Figure 2G**). The lesions with volumes less than 0.25 mm^3^ were overestimated by 2-fold when using 1 mm slice thickness, but gradually converged to similar volumes for the lesions of volume > 1 mm^3^. A Bland-Altman analysis of differences and agreement between the volumes showed that majority of lesion volumes were well within the lines of agreement (**Figure 2H**).

We further investigated whether 3-dimensional acquisition would have better sensitivity in detecting the smaller lesions. To do this we compared the SNR and acquisition time between 2D and 3D images acquired with 0.5 mm slice thickness equivalents. Representative 2D and 3D images with 0.5 mm slice thickness are shown in **Figure S1A**. **Figure S1B** lists the total acquisition time, signal to noise and contrast to noise ratio for 2D and 3D images. For the resolution that is required to detect small lesions, the 3D images took significantly more time and resulted in similar SNR and CNR compared to 2D. It is important to note that repetition time for 3D images was lower at 200 ms. The lesion volumes from 0.5 mm slice thickness images ranged between 1 – 3.1 mm^3^. The Pearson correlation analysis of the tumor volumes estimated from 2D versus 3D sequences showed a significantly positive correlation (**Figure S1C**). No additional lesions were observed in 3D acquisition images that was not present in the 2D acquisition images.

### Baseline Characterization of Diffusion and Perfusion Imaging

The mean ADC of lesions at baseline was 0.0019 ± 0.0006 mm^2^/s with the values ranging from 0.0007 to 0.0033. The smallest lesion for which we were able to calculate the ADC was 0.61 mm^3^. A histogram analysis of the values is given in **Figure S2**. The majority of the lesions (~73%) had ADC values higher than 0.0018 mm^2^/s. We observed no correlation between baseline ADC and lesion volumes or normalized T_2_ intensities (data not shown).

**Figure 3:**
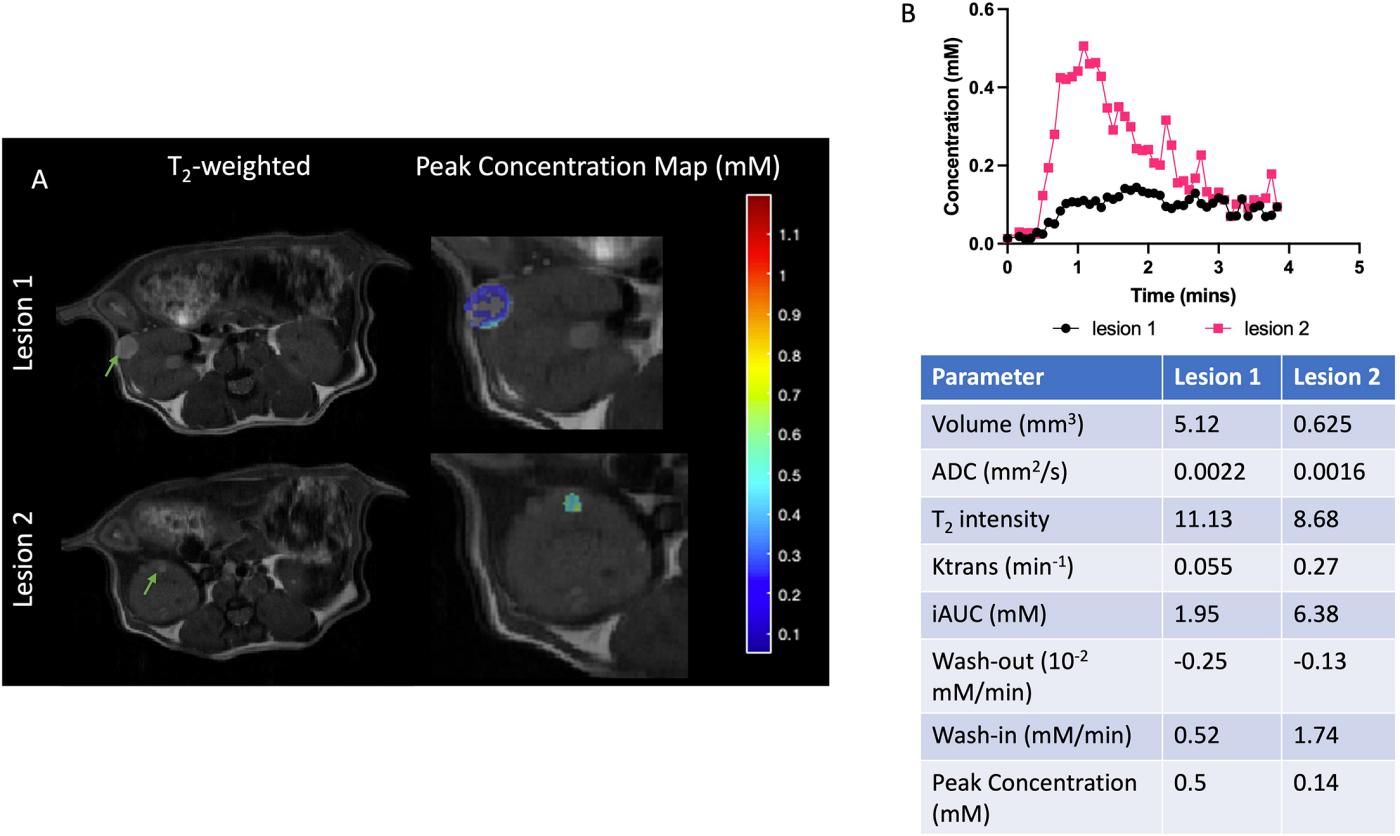
Representative cystic and cellular lesions and their baseline properties. **(A)** Representative T_2_-weighted and magnevist peak concentration maps. **(B)** Dynamic curves of magnevist concentration from two lesions. Table with physiological and DCE parameters for the lesions. Lesions are indicated with green arrows.

DCE analysis was performed on three mice at baseline and 21 lesions in total were identified. Two lesions representing the range of DCE parameters are shown in **Figure 3**. These selected lesions show the two distinct dynamic curves of the contrast agent and the corresponding physiological parameters and the mean and standard deviations of all the quantitative and semiquantitative parameters obtained of the representative lesions are tabulated in **Figure 3**. As shown in **Figure 3**, lesion 1 with higher volume had much slower wash-in of Gd-DTPA and peak concentration, compared to lesion 2 which showed a faster accumulation of the contrast agent and higher peak concentration. Lesion 2 also had faster wash-out and higher K^trans^, i.e., permeability. The concentration maps overlaid on the lesions show the lack of contrast agent build-up within the center of lesion 1. Lesion 1 with higher ADC of 0.0022 mm^2^/s had a significantly lower accumulation of the contrast agent as compared to lesion 2 with ADC of 0.0016 mm^2^/s which showed a rapid uptake and washout of the contrast agent. The concentration curves of all the individual lesions are shown in **Figure S3**.

**Figure 4:**
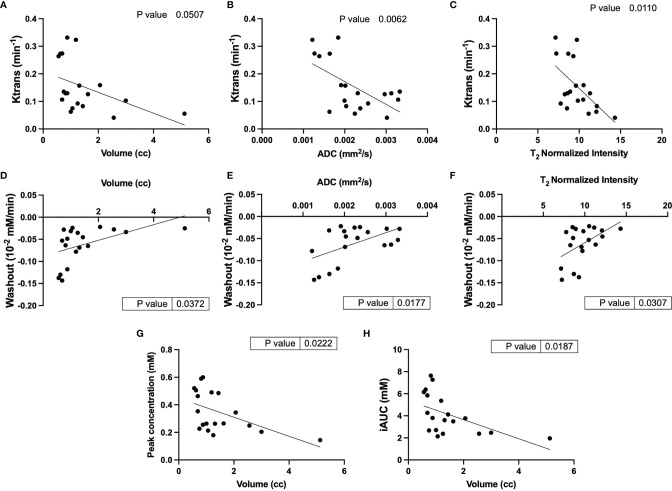
Correlational analysis of quantitative and semi-quantitative DCE parameters and physiological parameters. Correlation of Ktrans with **(A)** volume, **(B)** ADC and **(C)** T2 normalized intensity; Washout correlated to **(D)** volume, **(E)** ADC and **(F)** T2 normalized intensity; and volume correlation with **(G)** peak concentration and **(H)** iAUC.

Clear patterns of perfusion parameters were visible in the different manifestations of cyst and cystadenomas in concordance with other imaging parameters. Correlation analysis showed that lesions with larger volumes had significantly lower K^trans^ (**Figure 4A**), slower washout (**Figure 4D**), lower iAUC (**Figure 4G**) and peak concentration (**Figure 4H**) indicating perfusion-impaired lesions. K^trans^ was also found to be significantly lower for lesions with higher ADCs (**Figure 4B**) and T_2_ intensities (**Figure 4C**), representative of more cystic lesions (19). Washout was significantly faster for lesions with lower ADCs (**Figure 4E**) and T_2_ intensities (**Figure 4F**) indicating solid adenomas (20). All the remaining correlations are shown in **Figure S4**. Lesions with larger volume and higher T_2_ intensity had slower wash-in of Gd-DTPA although not significant. DCE-MRI enhancement pattern has been used to differentiate between truly cystic (no internal enhancement) and soft-tissue lesions (some internal enhancement). Lesion 1 in **Figure 3** is indicative of a cystic lesion.

Lesions with larger tumor volumes demonstrated much lower enhancement and permeability compared to smaller lesions. Lesions with higher ADCs and normalized T_2_ intensities had lower permeability and slower clearance of contrast agent representative of cystic lesions. It was observed that lesions with slower clearance also had slower uptake of the contrast agent, although this trend was not significant.

### Response of TSC Lesions to Treatment

**Figure 5:**
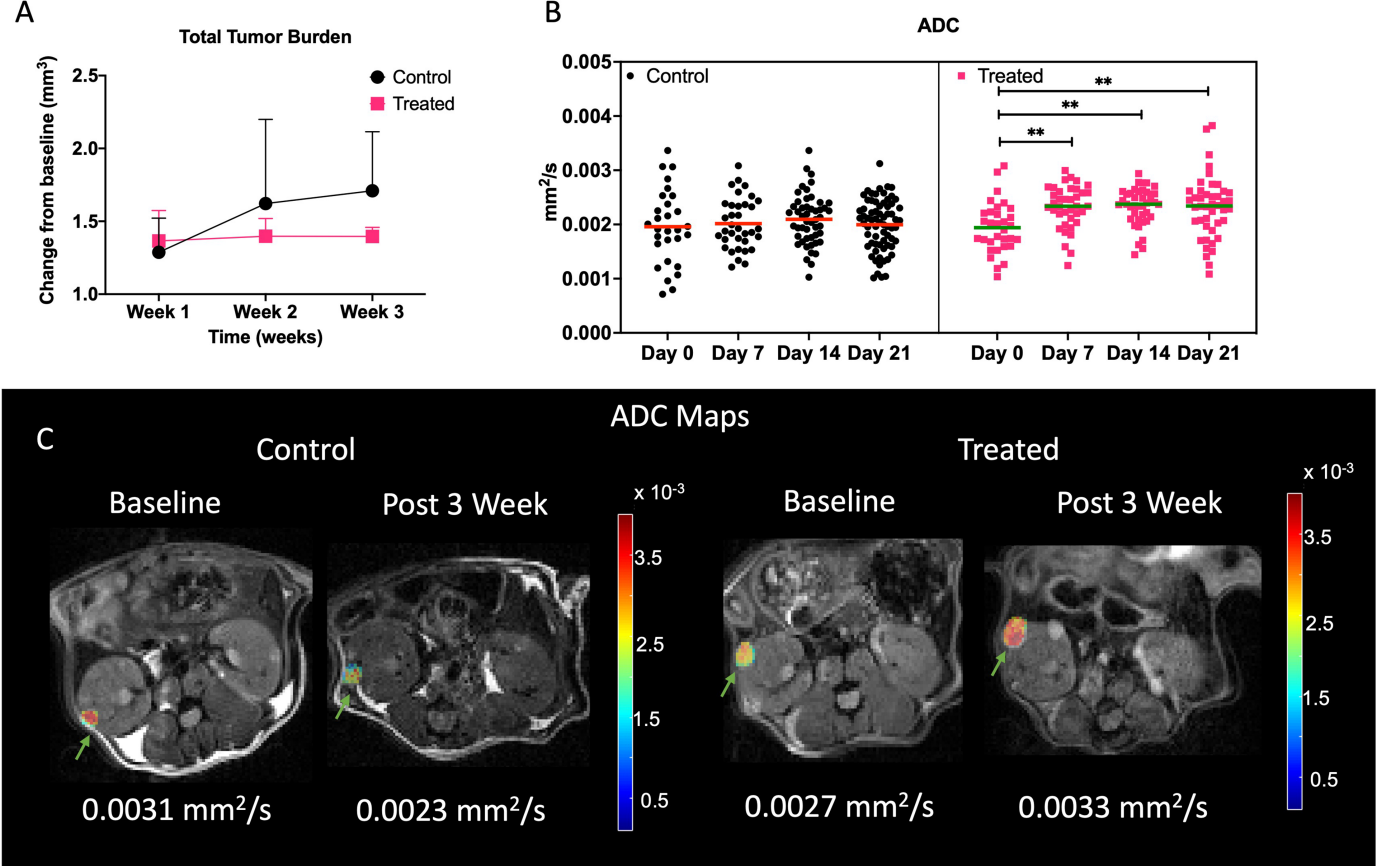
Impact of treatment on physiological properties of lesions. **(A)** Lesion volume change with respect to baseline in control and treated mice. **(B)** Mean ADC of all the lesions in control and treated cohort over time. Representative ADC maps overlaid on diffusion weighted imaged at *b* = 25 s/mm^2^ at baseline and week 3 for control and treated tumors **(C)**. Lesions are indicated with green arrows. ** indicates p<0.01.

We evaluated the changes in tumor volume to measure the effect of everolimus on *Tsc2^+/-^* mice. First, we compared the changes in total tumor burden from baseline in the two groups, everolimus-treated and untreated controls. On average it was observed that the tumors of mice treated with everolimus, after the initial increase in volume in the first week, did not demonstrate any further significant change in total tumor burden over the remaining three weeks (increased by 36 ± 20% by week 1, 39 ± 12% by week 2 and 39 ± 06% by week 4 compared to baseline) (**Figure 5A**). In contrast, tumors of mice in the control group increased in volume week over week (28 ± 23% by week 1, 62 ± 57% by week 2 and 71 ± 40% by week 4 compared to baseline). Mice treated with everolimus demonstrated a deceleration in total tumor burden compared to control, although a two-way ANOVA analysis showed that it was not statistically significant. **Supplementary Figure S5** demonstrates a representative example of change in volume for control and treated lesions and individual tumor burdens.

Mean tumor ADCs (**Figure 5B**) of treated mice increased over baseline at week 3 (2.3 ± 0.5 x 10^-3^ mm^2^/s) while tumors in control mice either showed a decrease or maintained similar mean ADCs as baseline (1.95 ± 0.7 x 10^-3^ mm^2^/s). One-way ANOVA analysis showed that ADCs were significantly different for treated cohort (p<0.001), and *post hoc* comparison showed that baseline ADC was lower than all the later time points (p<0.01). **Figure 5C** demonstrates a representative example of change in ADC for control and treated lesions. Increase in ADC for the treated lesion was observed whereas in control lesion a decrease in ADC was observed.

Closer analysis of the changes in lesions over time using linear mixed model analysis demonstrated a slight difference in the rates of change in ADC (p = 0.0836) and volumes (p = 0.0898), where ADC slightly increased and volumes slightly decreased for treated tumor while ADC slightly decreased, and volume slightly increased for control tumors (**Figure S6A, B**). **Figure S6C** shows the changes in T_1_ in control and treated mice. We observed an increase in T_1_ at week 3 (3.2 ± 0.85 secs) from baseline (2.97 ± 0.21 secs) in control mice and a decrease in T_1_ of treated mice at week 3 (2.16 ± 0.9 secs) from baseline (2.63 ± 0.4 secs). Treated and control tumors showed statistically significant differential changes in T_1_ (p = 0.0495), with treated tumors significantly decreasing (-0.0058/day) and control tumors significantly increasing (.0276/day) (p = 0.0272).

Normalized T_2_ intensity changes showed opposite trends for control and treated mice. In control mice the intensities at week 1 (10.2 ± 2.5) was slightly higher than baseline (9.75 ± 1.4, **Figure S6D**). Following week 1 the intensities decreased to 8.6 ± 1 by week 2 and to 7.5 ± 0.8 by week 3. In treated mice the intensities increased slightly from 10.1 ± 1.6 at baseline to 11 ± 3.5 at week 1. Following week 1 the intensities increased to 12.6 ± 0.4 at week 3. Treated and control tumors showed statistically significant differential changes in T_2_ intensities (p = 0.0004), with treated tumors significantly increasing (0.0841/day) and control tumors significantly decreasing

(-0.1058/day) (p = 0.0084).

This implies that with everolimus treatment the lesions had increased cystic characteristics and lesions in control group had higher cellular component. Studies have shown that papillary lesions tend to be hypointense (21) on T_2_-weighted images as compared to cystic lesions (22).

### Immunohistochemical Analysis of Lesions

**Figure 6:**
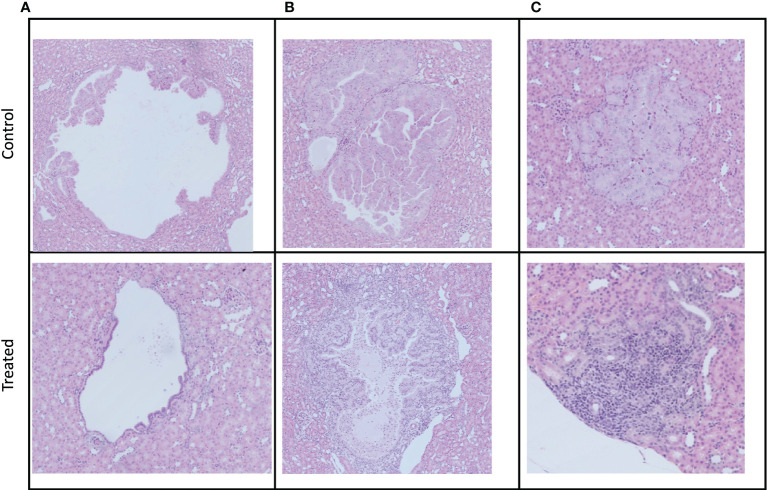
Examples of the three different manifestations of kidney cystadenomas in control and treated mice. Tumors were harvested after week 3 imaging. All pictures were taken at a 10× magnification. **(A)** Cystic lesions. **(B)** Papillary lesions. **(C)** Solid lesions.

**Figure 7:**
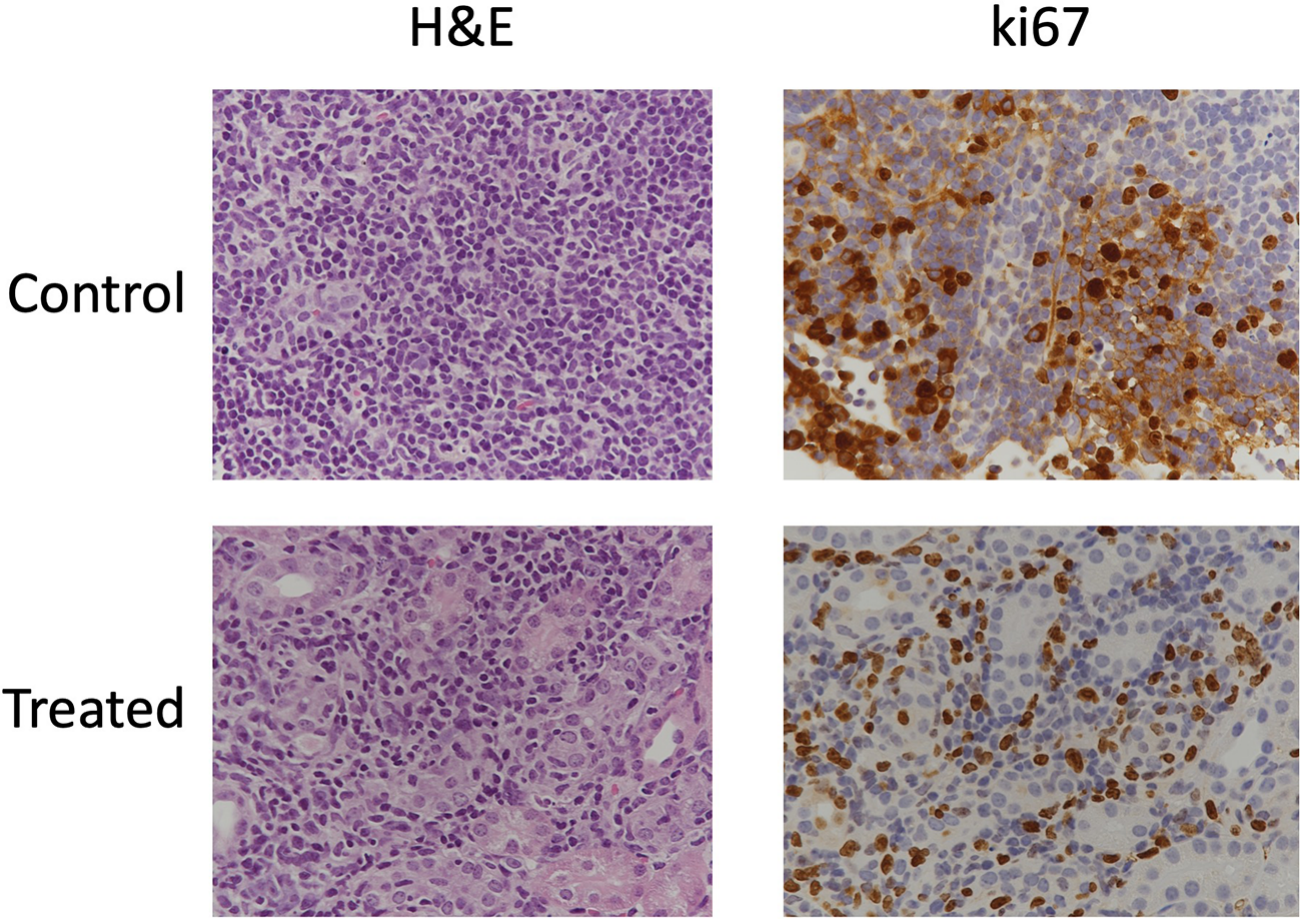
Immunohistochemical stains of cellularity (H&E) and proliferation (Ki67) in solid lesions in control and treated mice.

We observed cystic, papillary, and solid lesions in kidneys stained with H&E for both treated and control cohort (**Figure 6**). We imaged left kidneys for all the 6 mice and observed 26 cystic, 8 papillary and 9 solid lesions in control mice compared to 21 cystic, 2 papillary and 2 solid lesions in everolimus-treated mice. On average we observed more cystic lesions per kidney in treated mice (90% of total lesions) compared to control (61% of total lesions). Concomitantly, there were fewer solid lesions in the treated group. In control mice, we observed a similar distribution of solid (n = 9) and papillary lesions (n = 8) (figure not shown). We also examined the expression of Ki67 proliferation marker and histology by H&E staining to measure the cellularity of lesions. **Figure 7** shows the 40x magnification image of solid lesions stained with H&E and Ki67 antibody from treated and control groups. Qualitatively, we observed higher cellularity and proliferation in the control lesion in comparison to treated.

## Discussion

TSC is a lifelong disorder that affects approximately 2 million people worldwide (1). Although the majority of patients have a near-normal lifespan, the disease can cause high morbidity, decreased quality of life and mortality in 5-8% of patients (17). While the clinical manifestations of this disease vary among individuals, renal manifestations are one of the leading causes of deaths. Limited treatment options are available to patients with renal manifestations and primarily involve therapy with mTOR inhibitors and percutaneous embolization if bleeding is a risk (7). Patients must undergo lifelong therapy with surveillance as the preferred method for managing the disease in these patients (2).

The *Tsc2* ^+/-^ mouse model is known to have cystic disease starting from 6-12 months of age (9, 10, 18). In our study, we observed multiple lesions in both kidneys in 6–7-month-old mice. The lesions are termed cystadenomas and exhibit a spectrum of phenotypes, from pure cysts to cysts with papillary projections to solid adenomas. We observed similar phenotypes as demonstrated in the histopathological examination of lesions in the mice studied (**Figure 7**).

Our study identified optimal MR parameters for studying the size, distribution, and physiological properties of TSC lesions including cellularity, perfusion, and diffusion. In this work, MRI was shown to be an efficient modality for robustly identifying cysts as well as cystadenomas in 6-7-month-old *Tsc2* ^+/-^ mice. We observed good SNR and image quality for all scans. While we did not implement respiratory gating, we recognize that it might help eliminate motion artifacts and facilitate obtaining images with even higher SNR, especially considering the use of the vertical bore microimaging scanner used in this work. Similar to prior studies, T2-weighted images were used to localize the lesions which appeared as hyperintense regions indicative of a cystadenoma phenotype (23, 24). Woodrum et al. (10) observed an average of 13.00 ± 4.28 lesions/kidney in 5-month-old mice from IHC sections, while in our study we observed 18 ± 2.8 lesions/kidney. The slightly higher count could arise from the lesion count being performed only every 1mm in the tumor using IHC compared to the entire kidney region of the MRI images in our study.

Since change in lesion volumes is the main indicator of therapeutic response (RECIST criteria) it is critical to capture the distribution and size of lesions reliably. A 2D T_2_-weighted imaging sequence with a smaller slice thickness of 0.5 mm at either axial or coronal orientation was found to be optimal. Although theoretically (based on 0.5 mm slice thickness) lesions of 0.0055 mm^3^ volume should be detectable, 0.016 mm^3^, the smallest lesion detectable in our study, could very well represent our detection limits based on observer performance.

Determining the composition of kidney lesions is critical in understanding the phenotype and morphology of lesions. AMLs are prone to bleeding as they increase in size. Techniques such as ADC and DCE measurements can provide us with information regarding cellularity and perfusion/permeability of the lesions. As seen in IHC images, the lesions differ in cellular composition. We observed that based on ADC values (**Figure S2**), the cysts being defined as those lesions with ADC > 0.0022 mm^2^/s (25) comprised 19 out of 61 lesions, i.e., 31% were found to be cystic at baseline. Taouli et al. (26) measured the ADC of renal cell carcinoma (RCC) and benign lesions in kidney and found RCC to have a significantly lower ADC of 0.0014 mm^2^/s than the benign lesions. They also showed that papillary RCC had lower ADC of 0.0012 mm^2^/s than non-papillary RCC (0.0016 mm^2^/s). Based on the same metrics 15% of the lesions in the mice are indicative of papillary phenotype. The proportion of the cystic to papillary lesions as characterized by ADC seems higher than that estimated by histopathology (10). This indicates that a more thorough correlation of MRI characteristics with histological assessment is necessary to better interpret the imaging findings, which is beyond the scope of this study.

Prior studies have shown the utility of quantitative and semi-quantitative DCE-MRI parameters as imaging biomarkers for assessing tumor aggressiveness and predicting response to therapy. We captured distinct kinetics of the contrast agent in different lesions (**Figure 4** and **S3**). We also observed that lesions with higher ADC similar to lesion 1 had significantly lower contrast agent accumulation in the center of the lesions, demonstrating limited perfusion towards the center of the lesion characteristic of cystic lesions (19, 25). Tumors with higher K^trans^ have been associated with better response to treatment (27, 28), as this indicates higher permeability and results in efficient delivery of therapeutic drug. Congruent to this we observed a lesion (not shown) with lower K^trans^ of 0.04 (baseline) that increased in volume from 2.5 mm^3^ to 4 mm^3^ after everolimus therapy. Conversely, a lesion with K^trans^ of 0.33 (baseline) decreased in volume from 1.5 mm^3^ to 0.8 mm^3^ after everolimus therapy. A study by Sun et al. (29) showed that differences in signal intensity as a response to contrast agent can also differentiate between clear cell and papillary RCCs, with clear cell RCC showing higher signal intensity change. Everolimus has been reported in previous studies to have anti-angiogenic effects (30, 31) and thus implementation of DCE-MRI in the standard imaging protocol for TSC will be critical in evaluating its activity.

Only three mice were imaged at baseline with DCE MRI in this study. Due to the loss of several mice early on during DCE imaging, this scan was omitted from our study at later time points. This could be due to impaired kidney function complicated by repeated contrast agent administration. Further studies are required to validate this theory. Treatment-induced changes in perfusion are critical to assess therapeutic efficacy and in future studies DCE MRI will be implemented for the entire course of imaging. Pharmacokinetic DCE-MRI has been increasingly applied in quantitative scientific research and clinical practice as studies have shown the utility of DCE-MRI parameters to predict the efficacy of chemotherapy (32) and to image activity of anti-angiogenic drugs (33, 34) noninvasively.

Analysis of baseline imaging characteristics demonstrates the importance of interpreting the lesion morphology for accurate scoring of disease. Therapeutic response is mostly measured by the change in lesion size, but other factors such as change in cellularity, signal intensity and vascularization/perfusion could also aid in early prediction of therapeutic efficacy.

Studies of *Tsc2* ^+/-^ and other mouse models treated with rapamycin (an FDA-approved macrolide antibiotic that acts to inhibit the mTOR pathway, and analogs) have shown the ability to restore dysregulated mTOR signaling in cells with abnormal TSC1 and/or TSC2 (5, 10, 24, 35). However, these studies were disadvantaged by the lack of imaging follow-up to evaluate response over time to potentially adapt the therapeutic regimen and obtain an earlier assessment. We observed a 70% increase in total tumor burden in control mice from baseline over a 3-week period. On the other hand, the tumor burden in everolimus-treated mice increased by approximately 37% after one week of therapy and maintained that tumor burden for the remaining two weeks of therapy. This seems to indicate that after the initial effect, the drug might play more of a maintenance role in suppressing the mTORC1 pathway. Pollizzi et al. (36) observed a 7-fold reduction in tumor burden in *Tsc2* ^+/-^ mice treated with N-ethyl-N-nitrosourea to increase the incidence and severity of renal lesions after treating with 10 mg/kg everolimus PO QD (5/7 days per week). Of note, the age of mice in the study by Pollizzi et al. was 20 months, perhaps relevant to the lesser magnitude of change in the younger mice in our study.

In another study, treatment of A/J *Tsc2* ^+/-^ mice (9 months old) with 8 mg/kg of rapamycin weekly for 12 weeks, or daily for 4 weeks and weekly for another 8 weeks, had similar effects in reducing tumor burden (~80%) indicating that duration of treatment is more important than dose in eliciting a significant response (10). Interestingly, everolimus treatment in patients showed a significant decrease in AML after 3 months of treatment and a higher reduction upon 3-6 months of treatment (37). Thus, while we observed modest changes over 3 weeks of treatment in our study, continued treatment with everolimus could have resulted in a higher decrease in tumor burden, considering that our treatment was also initiated in younger mice than in prior studies. It is important to note that studies have shown that, in *Tsc2* ^+/-^ mice, therapeutic intervention demonstrates reduction in tumor volumes, but the cessation of therapy results in recovery of tumor growth (10, 14, 35), highlighting the importance of prolonged treatment and monitoring. As stated earlier, this was a pilot study evaluating the feasibility of incorporating mp-MRI for studying disease progression and treatment response in this transgenic mouse model. The sample size for this study was low and for future studies a larger cohort of mice per cohort should be evaluated. We recommend exploring different dosing range and schedule in TSC models with a larger sample size to determine the optimum dosage of everolimus to observe significant change in tumor volume.

In addition to tumor volume changes, the gold standard of response criteria, we investigated if mpMRI, such as ADC, T_2_ weighted intensity and T_1_, could inform on therapeutic efficacy earlier. Normalized T_2_ intensity can be a useful measure for understanding the composition of a tumor. Studies have shown that different types of renal lesions show different signal properties. For example, lesions with fluid, edema, or impaired blood flow will appear hyperintense on a T_2_ weighted image (21). In our study, we observed a decrease in T_2_ intensity in control mice and an increase in everolimus-treated mice. This difference was even more significant when examined on a lesion-specific basis over time. We believe that this could be due to the anti-angiogenic effect of everolimus. In contrast, the lesions in control mice could become more cellular as they proliferate. The ADC changes in both the groups seem to validate this hypothesis, as we observed an increase in mean ADC post-everolimus treatment and a decrease in control mice. This demonstrates a decrease in cellularity, a hallmark of treatment efficacy (38). While ADC could provide an earlier indicator than volume changes, we were limited by the number of lesions we could track over time in our study. Diffusion weighted images were acquired at 1 mm slice thickness which could have resulted in our sequences not capturing lesions that were less than 0.5 mm in diameter after therapy. Interestingly, we observed three lesions that had resolved entirely after three weeks of therapy only in everolimus-treated mice. T_1_ relaxation times have been shown to be an unreliable metric for differentiating between the types of lesions (39). The change in T_1_ (ΔT_1_) on the other hand has been shown to indicate response to therapy (40). That study showed that everolimus-treated (10 mg/kg for 7 days) RIF-1 fibrosarcoma- and B16/BL6 melanoma- bearing mice showed significant decreases in T_1_ post-therapy. We observed a similar trend in our everolimus-treated mice, indicating that ΔT_1_ can be a highly sensitive predictor of response to treatment.

Histopathological analysis of lesions after 3 weeks of treatment showed characteristic renal cystadenomas in both everolimus-treated and control mice (**Figure 7**). A higher percentage of papillary lesions (18%) and solid lesions (21%) was found in control kidney as compared to 8% of papillary and solid lesions in everolimus -treated kidney. A significantly lower tumor burden from papillary and solid cystadenomas in treated mice seems to indicate a specific everolimus effect on these adenomas. Auricchio et al. showed that the cystic lesions in rapamycin-treated mice had distinct reduction in cyst-lining cells as compared to control mice (12). In our study, too, we observed in the IHC images that cystic lesions of treated mice had a thinner lining of cells as compared to that in control mice (**Figure 7**). A more comprehensive analysis of IHC images and correlation with MR images at baseline would be invaluable in assessing specific effects of therapy on these different lesions non-invasively over time and useful for development of combinatorial targeted therapy.

A qualitative comparison of cellularity and proliferation from IHC images between solid cysts from both the cohorts showed higher cellularity and proliferation in control mice. In future, quantitative analysis of these IHC parameters along with ADC and tumor volume could provide better assessment of treatment response and mechanism of drug action. Evaluation of IHC markers associated with mTORC1 such as phospho-S6 (pS6), hypoxia-inducible factors (HIFs) and vascular endothelial growth factors (VEGFs) can inform upon the efficacy of its inhibition. Expression of pS6, HIF and VEGF are upregulated in Tsc2+/- models and studies have shown their reduction after treatment with mTOR inhibitors (30, 36, 41–43).

Previous studies have shown that combination treatments such as sorafenib and mTOR pathway inhibitors are also effective in *Tsc2* ^+/-^ mouse models (10, 14, 24, 36). The utility of mp_MRI in evaluating cancer models such as renal cancer (44, 45), prostate cancer (46) and breast cancer (47) have been reported before. Future preclinical studies using *Tsc2* ^+/-^ and other TSC mouse models with mpMRI offer a rational approach to improving medical therapy for TSC-related tumors and other manifestations of TSC.

## Conclusion

There is a critical need for development of non-invasive imaging strategies for TSC-derived tumor lesions to monitor the progression and relapse of the disease upon treatment. The current study shows the ability of multiparametric ^1^H MRI in providing vital information regarding the tumor’s characteristics non-invasively, thus allowing for a dynamic evaluation of the disease progression and treatment response. Our results show that high resolution 2D T_2_-weighted images with thinner (0.5 mm) slice thickness can be used to capture tumor growth robustly. ADC maps showed that cellularity of tumor lesions with diameter less than 0.75 mm in size can be evaluated and can also be used to investigate the changes in cellularity as a response to therapy. Our study has laid the groundwork to use non-invasiveMRI to characterize the various renal manifestations of TSC and can be used to evaluate response to therapy since different lesions might respond differently to different treatments and can ultimately help tailor therapy.

## Data Availability Statement

The original contributions presented in the study are included in the article/**Supplementary Material**. Further inquiries can be directed to the corresponding author.

## Ethics Statement

The animal study was reviewed and approved by UCSF IACUC.

## Author Contributions

SA performed the experiments, data analysis and drafted and edited the manuscript. ED-B assisted in performing imaging of mice and data analysis. Y-HW assisted in dosing and experiments. RDS performed the IHC staining of kidneys. HQ assisted in DCE modeling, statistical analysis as well as in critical evaluation. ME provided funding, critical guidance for the experiments and manuscript. RS provided funding, experimental/study design, critical guidance for the experiments, and was responsible for supervising the execution of the study, writing and editing of the manuscript. All authors have read and approved the final manuscript.

## Funding

ME was supported by the American Cancer Society (30635-RSG-17-005-01-CCE) and a pilot grant from the LAM Foundation (LAM0143P01-20).

## Conflict of Interest

The authors declare that the research was conducted in the absence of any commercial or financial relationships that could be construed as a potential conflict of interest.

## Publisher’s Note

All claims expressed in this article are solely those of the authors and do not necessarily represent those of their affiliated organizations, or those of the publisher, the editors and the reviewers. Any product that may be evaluated in this article, or claim that may be made by its manufacturer, is not guaranteed or endorsed by the publisher.

## Supplementary Material

Supplementary Figure 1 Comparison between lesions captured *via* 2D and 3D image acquisition. **(A)** Representative 2D and 3D images of the kidney acquired at 0.5 mm slice thickness. Lesions are indicated with green arrows. **(B)** Comparison between image quality of 2D and 3D images. **(C)** Comparison of 2D vs 3D at 0.5 mm slice thickness lesion volumes.

Supplementary Figure 2 Histogram analysis of ADC values of lesions at baseline.

Supplementary Figure 3 Dynamic curves of contrast agent concentration in all the lesions at baseline.

Supplementary Figure 4 Correlational analysis of DCE parameters and physiological parameters.

Supplementary Figure 5 Representative T_2_-weighted images of lesions at baseline and week 3 for control and treated tumors. Lesions are indicated with green arrows. Line graphs showing individual tumor burdens for untreated control and Everolimus treated mice.

Supplementary Figure 6 Lesion-wise changes in **(A)** ADC **(B)** volumes, **(C)** T_1_ and **(D)** normalized T_2_ intensity of control and treated mice.
