# Supplementary figures and images for: Defining the Magnetic Resonance Features of Renal Lesions and Their Response to Everolimus in a Transgenic Mouse Model of Tuberous Sclerosis Complex
Source: Front Oncol. 2022 Jun 23;12:851192. doi: 10.3389/fonc.2022.851192 (PMC9260108; doi:10.3389/fonc.2022.851192)

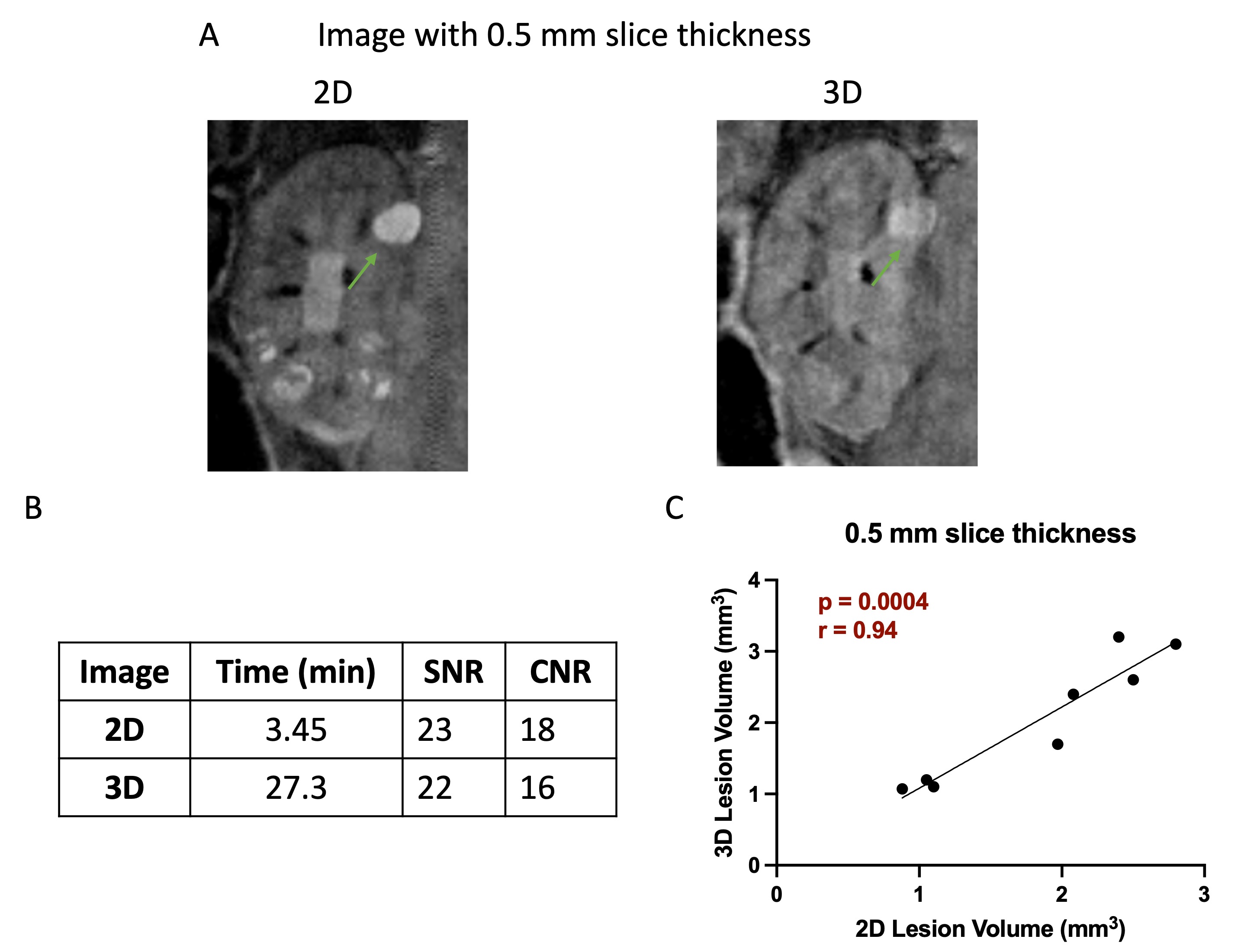

Supplement: Supplementary Figure 1 — Comparison between lesions captured via 2D and 3D image acquisition. (A) Representative 2D and 3D images of the kidney acquired at 0.5 mm slice thickness. Lesions are indicated with green arrows. (B) Comparison between image quality of 2D and 3D images. (C) Comparison of 2D vs 3D at 0.5 mm slice thickness lesion volumes. [file Image_1.jpeg]

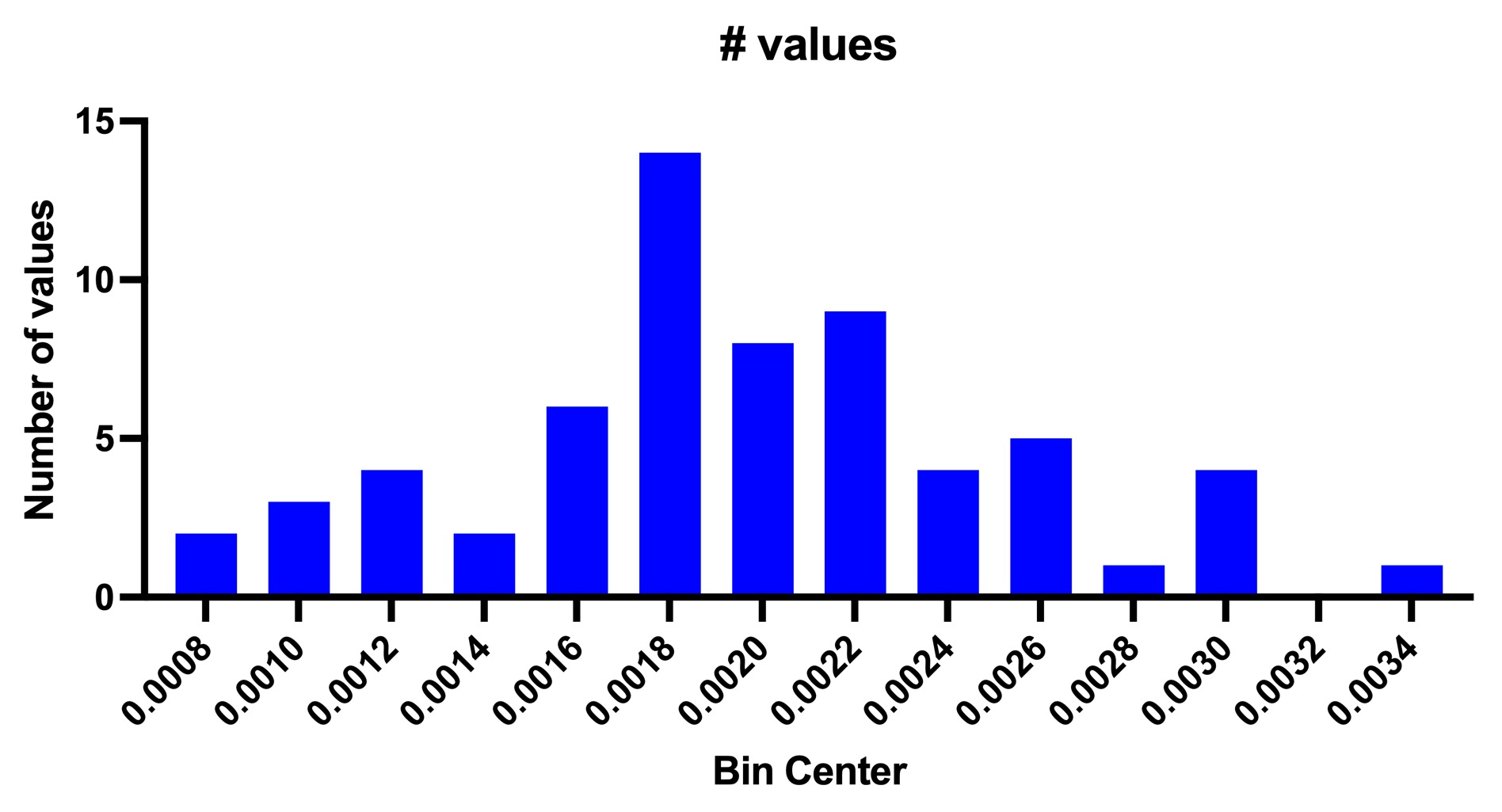

Supplement: Supplementary Figure 2 — Histogram analysis of ADC values of lesions at baseline. [file Image_2.jpeg]

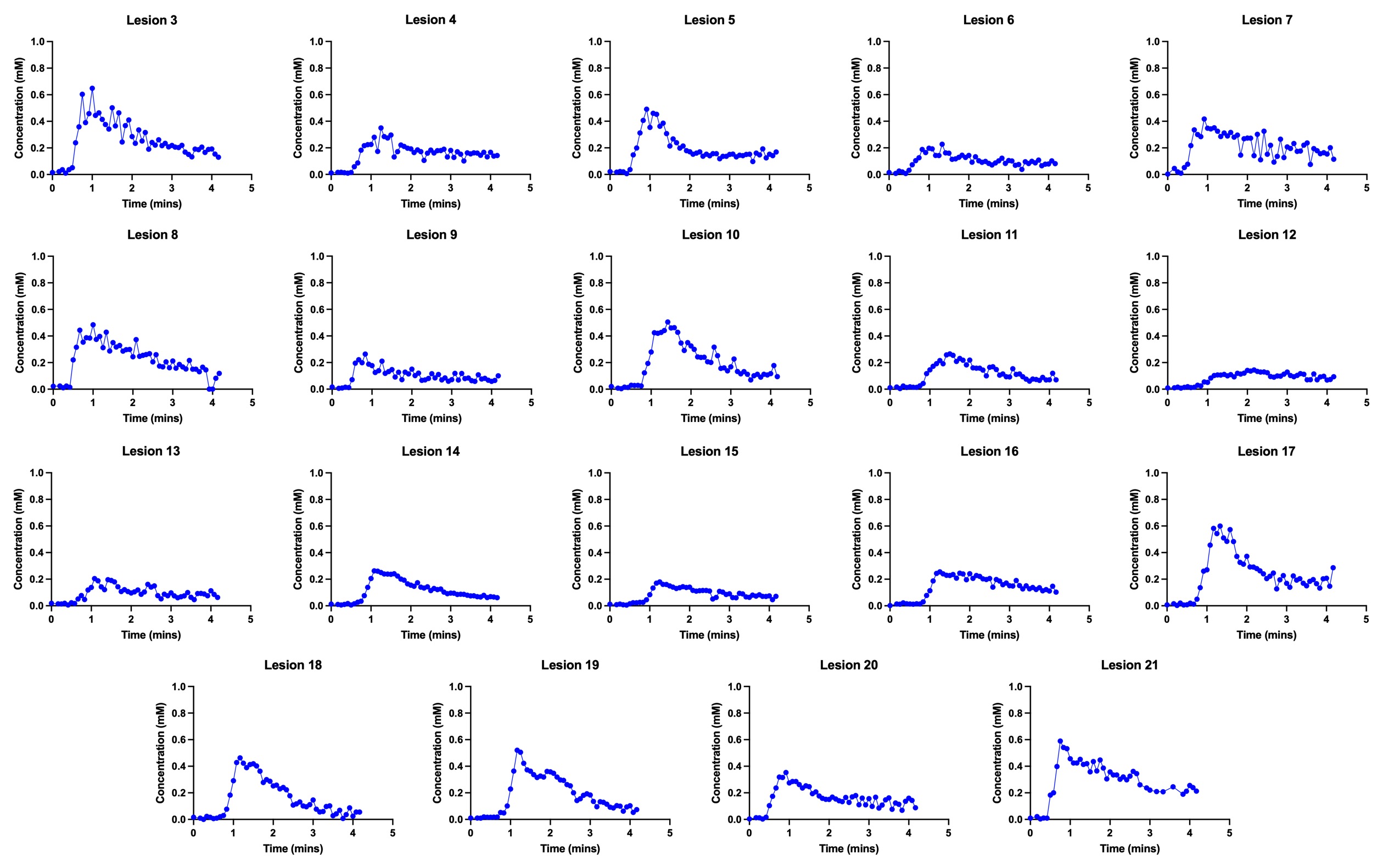

Supplement: Supplementary Figure 3 — Dynamic curves of contrast agent concentration in all the lesions at baseline. [file Image_3.jpeg]

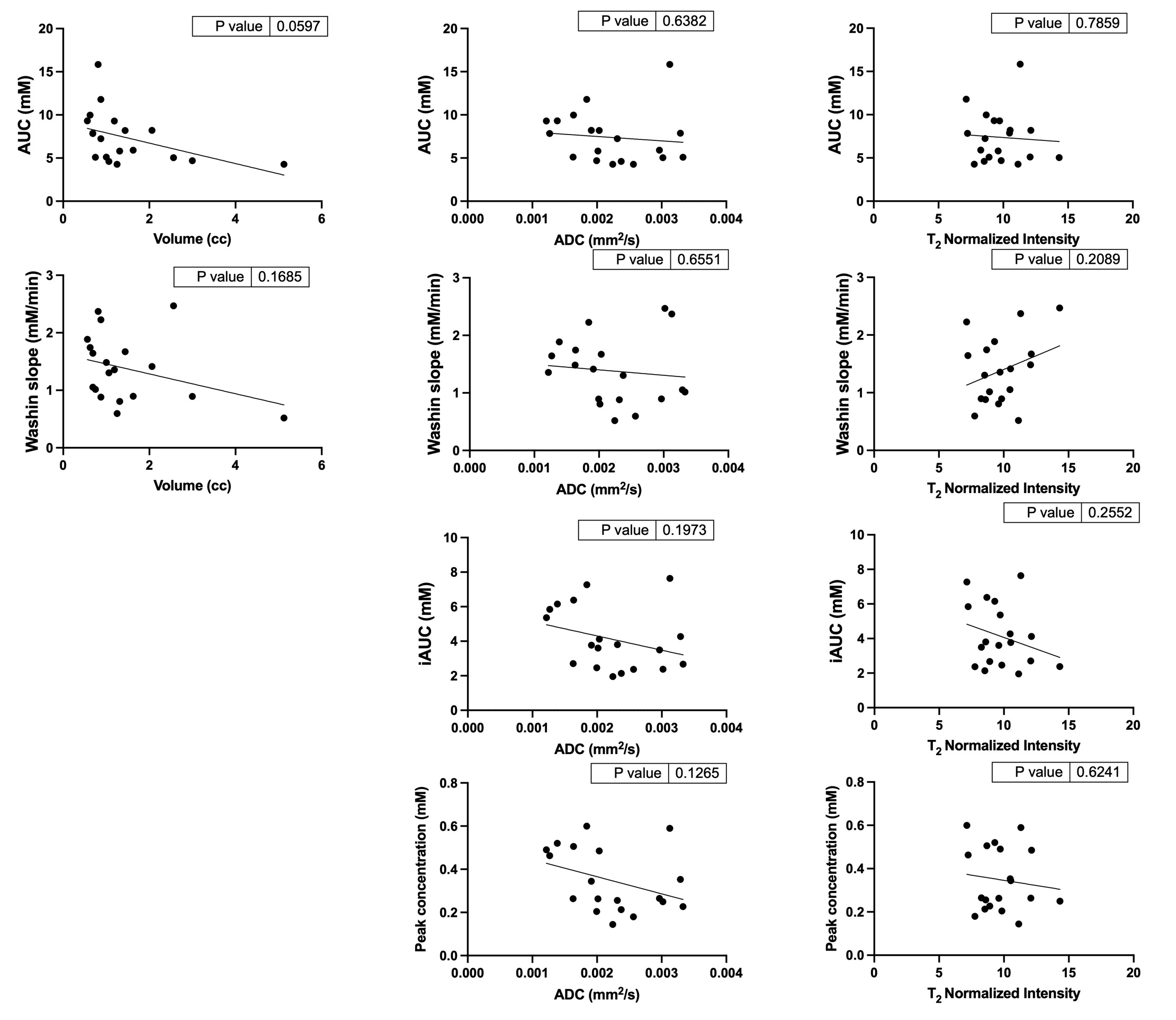

Supplement: Supplementary Figure 4 — Correlational analysis of DCE parameters and physiological parameters. [file Image_4.jpeg]

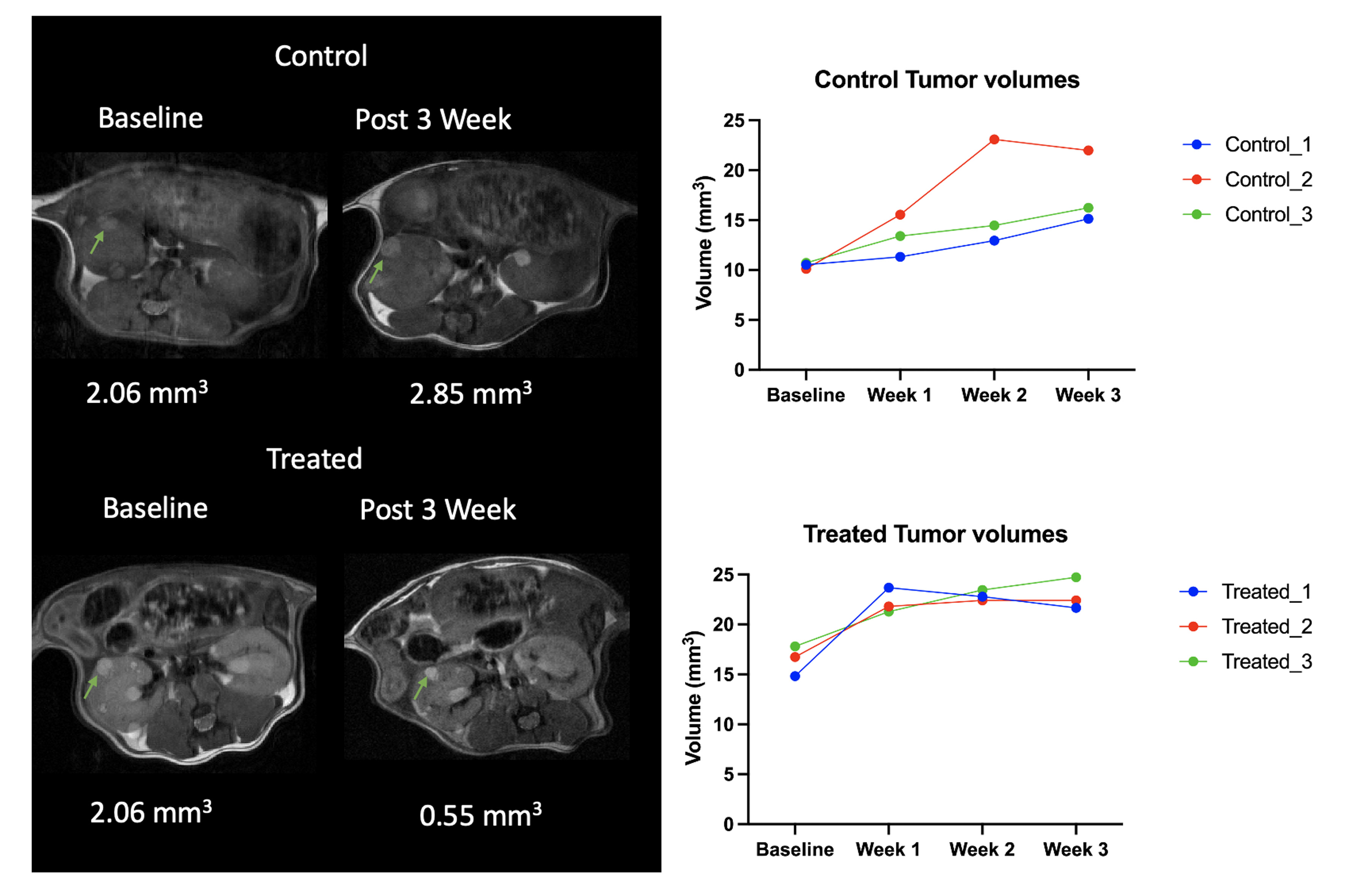

Supplement: Supplementary Figure 5 — Representative T2-weighted images of lesions at baseline and week 3 for control and treated tumors. Lesions are indicated with green arrows. Line graphs showing individual tumor burdens for untreated control and Everolimus treated mice. [file Image_5.jpeg]

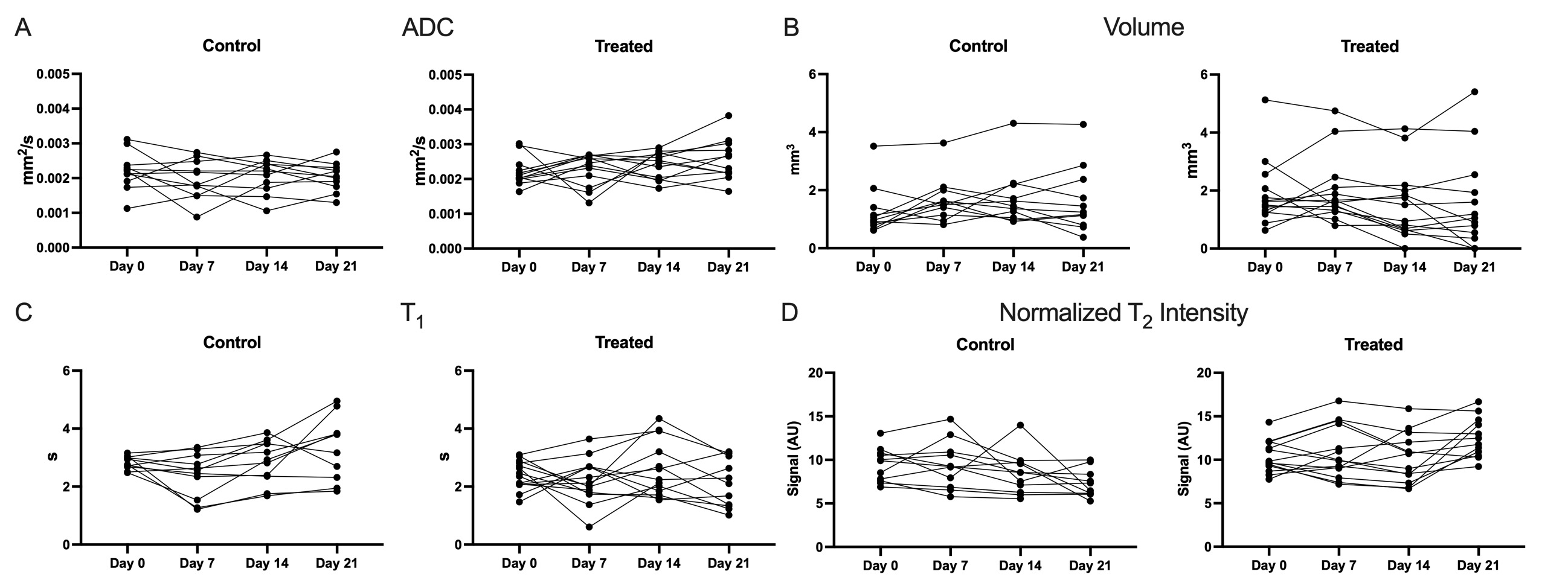

Supplement: Supplementary Figure 6 — Lesion-wise changes in (A) ADC (B) volumes, (C) T1 and (D) normalized T2 intensity of control and treated mice. [file Image_6.jpeg]
